# Immune Checkpoint Inhibitor Data in Metastatic Melanoma with Pre-Existing Autoimmune Disease: A Real World Experience

**DOI:** 10.3390/ijms27198832

**Published:** 2026-10-03

**Authors:** Maria Grazia Vitale, Domenico Mallardo, Francesca Sparano, Lucia Festino, Vito Vanella, Claudia Trojaniello, Margaret Ottaviano, Eleonora Cioli, Mario Mallardo, Bianca Arianna Facchini, Ester Simeone, Paolo Antonio Ascierto

**Affiliations:** 1 Melanoma, Cancer Immunotherapy and Development Therapeutics Unit, Istituto Nazionale Tumori-IRCCS-Fondazione “G. Pascale”, 80131 Naples, Italy; 2 Dermatology Unit, Department of Medical and Surgical Sciences, Fondazione Policlinico Universitario A. Gemelli IRCCS, 00168 Rome, Italy; 3 Department of Neurosciences, Reproductive Sciences and Odontostomatology, University of Naples Federico II, 80131 Naples, Italy

**Keywords:** melanoma, autoimmune disease, immune checkpoint inhibitors, PD-1, immune-related adverse events, real-world evidence

## Abstract

Patients with pre-existing autoimmune disease (AID) are underrepresented in trials of immune checkpoint inhibitors (ICIs). We retrospectively compared 41 patients with stage IV melanoma and pre-existing AID with 162 contemporaneous controls without AID who received ICI treatment between November 2016 and April 2023. In the AID and control cohorts, respectively, the objective response rates were 46.3% and 32.7%, and the disease control rates were 80.5% and 51.2%. Treatment interruption attributed to worsening of the baseline AID was recorded in 11/41 patients, while 13/41 interrupted treatment because of toxicity distinct from the baseline AID. The full cohort contributed 114 progression-free survival (PFS) events and 92 deaths. In models adjusted for age, sex, ICI regimen and M category, AID was associated with PFS (adjusted hazard ratio [HR] 0.48, 95% confidence interval [CI] 0.24–0.99; *p* = 0.046), whereas the association with overall survival (OS) was not statistically significant (adjusted HR 0.63, 95% CI 0.29–1.34; *p* = 0.232). These findings support the feasibility of ICI therapy in carefully selected patients with clinically stable AID, while the observational design and residual confounding require cautious interpretation of comparative outcomes.

## 1. Introduction

Immune checkpoint inhibitors (ICIs) have transformed the treatment of advanced melanoma. Anti-PD-1 antibodies such as nivolumab and pembrolizumab produce durable responses and improve survival compared with earlier systemic therapies [1,2]. The combination of nivolumab and the anti-CTLA-4 antibody ipilimumab provides additional activity at the cost of greater immune-related toxicity, with durable outcomes confirmed in long-term follow-up of CheckMate 067 [3]. Nivolumab plus relatlimab has further expanded first-line options through dual PD-1 and LAG-3 blockade [4].

Patients with pre-existing AID were largely excluded from pivotal ICI trials. Retrospective melanoma series and studies in non-small-cell lung cancer and mixed solid tumors suggest that treatment can be feasible, but estimates of flare risk and efficacy vary because cohorts differ in autoimmune phenotype, baseline activity, immunosuppressive treatment, tumor type and ICI regimen [5,6,7,8,9,10,11,12,13,14,15,16,17,18]. Direct comparisons of OS and PFS across these populations are therefore difficult. Published studies evaluating ICIs in patients with pre-existing AID are summarized in Supplementary Table S1.

Important uncertainties remain regarding the effect of baseline prognostic imbalance, the risks associated with individual autoimmune conditions and the interpretation of survival differences between patients with and without AID. Many published series lack a contemporaneous melanoma control group or provide limited multivariable adjustment.

We therefore compared patients with stage IV melanoma and pre-existing AID with a contemporaneous control cohort without AID. The analysis describes treatment interruptions related to worsening of the baseline AID or other toxicity, formally compares baseline characteristics and evaluates PFS and OS after adjustment for prespecified prognostic factors.

## 2. Results

### 2.1. Patient Characteristics

The analysis included 41 patients with pre-existing AID and 162 controls without AID. All patients had AJCC stage IV melanoma at ICI initiation. Patients with AID were younger than controls (median age 54 vs. 66 years; *p* < 0.001), while sex distribution did not differ materially. The overall M-category distribution differed between cohorts (M1a/M1b/M1c/M1d: 3/14/22/2 vs. 36/33/74/19; Fisher exact *p* = 0.037), although M1c-M1d disease was similarly represented (58.5% vs. 57.4%; *p* = 1.000). BRAF mutations were reported in 2/41 AID patients and in no controls (*p* = 0.040). Anti-PD-1 monotherapy was administered to 31/41 AID patients and all 162 controls; 10/41 AID patients received a CTLA-4-containing regimen (Table 1; Supplementary Table S2). None of the AID patients was receiving systemic immunosuppression at ICI initiation, and no patient had received a disease-modifying antirheumatic drug during the preceding six months. The distribution of autoimmune diagnoses is presented in Table 2. Supplementary Tables S3–S7 provide the exploratory AID-only Cox models, full-cohort Cox models, OS sensitivity analyses, comparative response estimates and treatment-interruption data, respectively.

### 2.2. Immunotoxicity

The retrospective database contained patient-level indicators of treatment interruption attributed to worsening of the pre-existing AID and of interruption for toxicity distinct from the baseline AID. These were recorded in 11/41 (26.8%) and 13/41 (31.7%) patients, respectively (Supplementary Table S7). The source data did not provide a complete, uniformly adjudicated event-level registry of flare and de novo immune-related adverse events with organ, grade, onset date, corticosteroid dose and permanent-discontinuation status. Corresponding granular toxicity data were not available for the control cohort.

### 2.3. Antitumor Activity

In the AID cohort, the objective response rate (ORR) was 46.3% (95% CI 32.1–61.3) and the disease control rate (DCR) was 80.5% (95% CI 66.0–89.8). In controls, ORR was 32.7% (95% CI 26.0–40.3) and DCR was 51.2% (95% CI 43.6–58.8). The corresponding risk ratios were 1.42 (95% CI 0.95–2.11; Fisher exact *p* = 0.143) for ORR and 1.57 (95% CI 1.27–1.94; *p* < 0.001) for DCR (Supplementary Table S6).

### 2.4. Survival

**Figure 1:**
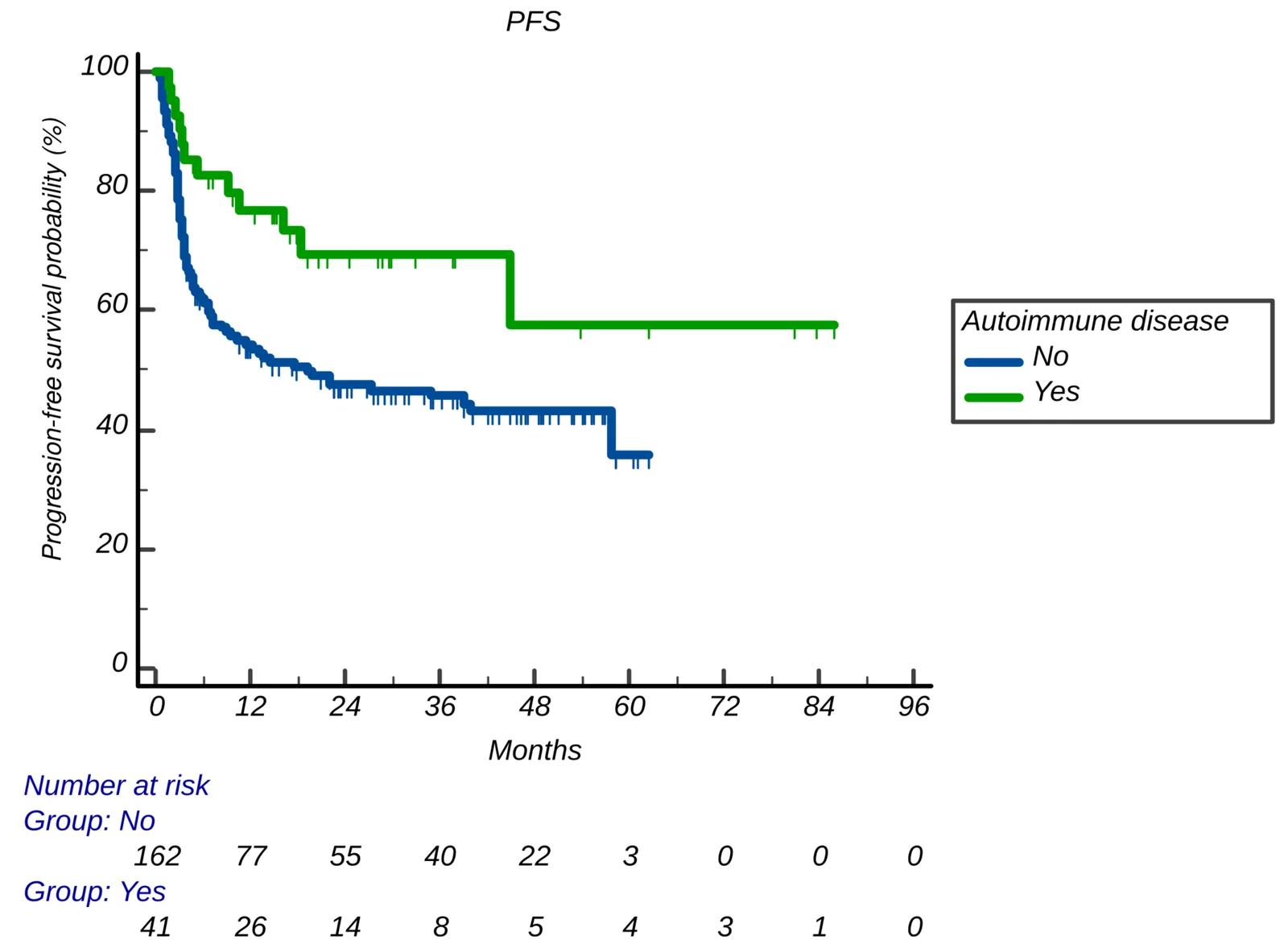
Progression-free survival according to pre-existing autoimmune disease status. Kaplan–Meier estimates of PFS from first ICI administration to radiological progression or death from any cause. Tick marks indicate censored observations; numbers at risk are shown below the plot.

**Figure 2:**
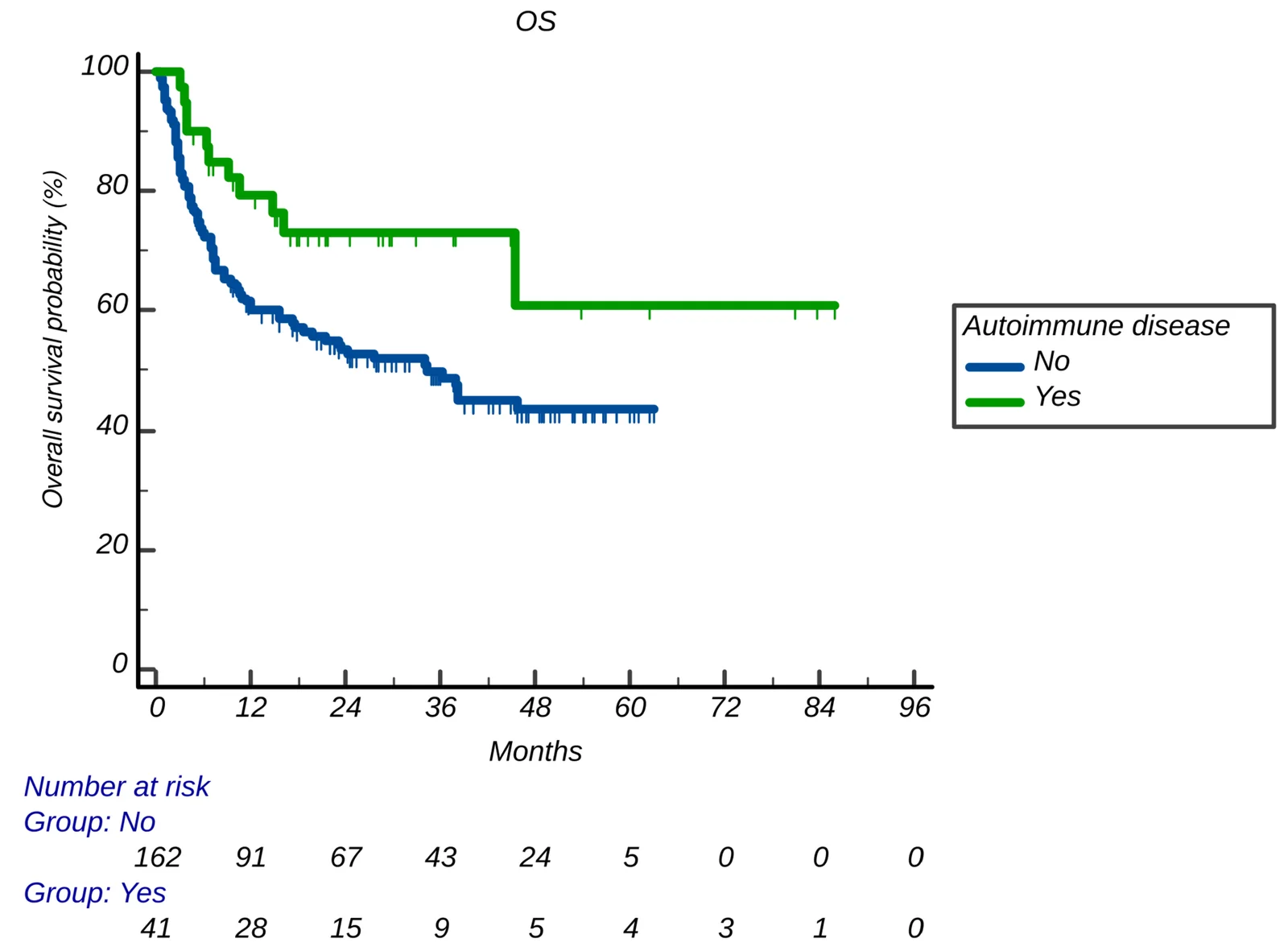
Overall survival according to pre-existing autoimmune disease status. Kaplan–Meier estimates of OS from first ICI administration to death from any cause. Tick marks indicate censored observations; numbers at risk are shown below the plot.

The full cohort contributed 114 PFS events and 92 deaths. In unadjusted analyses, AID was associated with longer PFS (HR 0.43, 95% CI 0.24–0.77; *p* = 0.005) and OS (HR 0.50, 95% CI 0.26–0.93; *p* = 0.030). After adjustment for age, sex, ICI regimen and M category, the association with PFS remained statistically significant (adjusted HR 0.48, 95% CI 0.24–0.99; *p* = 0.046), whereas the association with OS was attenuated and no longer statistically significant (adjusted HR 0.63, 95% CI 0.29–1.34; *p* = 0.232) (Figure 1 and Figure 2; Supplementary Table S4).

Age was the principal source of attenuation of the OS association: adjustment for age alone yielded an AID HR of 0.63 (95% CI 0.33–1.21; *p* = 0.169), whereas a model adjusted for sex and regimen without age yielded an HR of 0.46 (95% CI 0.22–0.96; *p* = 0.038) (Supplementary Table S5). Within the AID cohort, 13 PFS events and 11 deaths were observed; AID-only and disease-specific analyses were therefore considered exploratory (Supplementary Table S3). No material departure from the proportional hazards assumption was detected.

## 3. Discussion

In this selected retrospective cohort, ICI treatment was feasible in patients with clinically stable pre-existing AID. Treatment interruptions related to worsening of the baseline AID or other toxicity remained clinically relevant. The adjusted analysis showed an association between AID and PFS, but the crude OS association was attenuated after adjustment and was no longer statistically significant. These data therefore do not support interpreting AID itself as a favorable prognostic factor.

Our findings are consistent with retrospective melanoma and mixed-tumor cohorts showing that patients with pre-existing AID can experience autoimmune flares and de novo immune-related adverse events while retaining antitumor activity [6,7,8,9,10,11,12,13,14,15,16,17,18]. Differences in case selection, baseline disease activity and immunosuppressive treatment limit direct comparison across studies.

Clinical decisions should consider the autoimmune diagnosis and organ at risk, current activity and recent flare history, baseline corticosteroid or immunosuppressive requirements, melanoma burden and prognostic features, and the expected benefit and toxicity of the proposed ICI regimen. Patients with active or organ-threatening AID require particular caution and multidisciplinary assessment.

Regimen selection was not randomized or protocolized, and the clinical rationale for each treatment choice was not consistently documented. We therefore cannot attribute CTLA-4-containing treatment to a specific disease characteristic. Regimen was included in the adjusted survival models because of its potential association with both efficacy and toxicity.

Published data suggest that rheumatologic AID, particularly rheumatoid arthritis, and inflammatory bowel disease warrant close monitoring because clinically relevant flares or gastrointestinal toxicity may occur [6,12,13,14]. Within the present cohort, the available data did not support reliable ranking of flare risk by autoimmune subtype.

The biological relation between pre-existing autoimmunity and ICI-induced immune activation remains incompletely defined. Melanoma-associated vitiligo illustrates a link between autoimmunity and antitumor immunity [19], while a separate study examined diabetes and outcomes with nivolumab plus relatlimab [20]. Studies of circulating immune cells have also reported associations between immune-related adverse events, immune-cell phenotypes and therapeutic antibody binding [21,22,23]. IL-17/Th17-related pathways have been implicated in autoimmune disease [24,25,26,27]. In melanoma, Váraljai et al. linked IL-17/Th17 activity to clinical benefit from dual CTLA-4 and PD-1 blockade [28]. These observations provide biological context; cytokines and immune-cell phenotypes were not measured in the present cohort and cannot explain its observed clinical outcomes.

Prospective studies should incorporate standardized disease-specific activity measures, complete event-level toxicity data, longitudinal treatment exposure and immune profiling. Such data are needed to identify patients at greatest risk of severe toxicity and to separate treatment effects from selection and prognostic factors.

The absence of systemic immunosuppression at ICI initiation defines an important aspect of the population studied. It also introduces selection bias and limits generalizability to patients with active AID or those requiring ongoing systemic immunosuppression.

Taken together, the results support a risk-adapted approach rather than routine exclusion of patients with controlled AID from ICI treatment. Careful selection, counseling and coordination between oncology and the relevant organ specialist remain essential.

This study has several limitations. It was retrospective and conducted at a single center, and the AID cohort was small, clinically heterogeneous and supported by only 13 PFS events and 11 deaths. Accordingly, AID-only and disease-specific analyses are exploratory. Although the primary models were adjusted for age, sex, ICI regimen and M category, residual confounding remains possible. Metastatic-site burden was unavailable for controls, and only two BRAF mutations were reported, both in the AID cohort, precluding stable adjustment for these variables. The selected population included clinically stable AID without systemic immunosuppression at ICI initiation, which limits generalizability to patients with active or organ-threatening disease. Dates and event-level details for flare, de novo immune-related adverse events and additional immunosuppression were not consistently recorded; these post-baseline exposures could not be modeled as time-dependent covariates or interpreted causally. Cycle-level treatment exposure, longitudinal LDH measurements, corticosteroid agent and dose, and disease-specific AID activity scores were also not systematically available.

## 4. Materials and Methods

### 4.1. Design and Setting

We conducted a single-center retrospective observational cohort study at Istituto Nazionale Tumori IRCCS Fondazione G. Pascale, Naples, Italy. Patients treated between November 2016 and April 2023 were included. The study was approved by the institutional Ethics Committee (protocol 37/22), and written informed consent was obtained in accordance with the approved protocol.

### 4.2. Patients

Eligible patients were adults with histologically confirmed stage IV melanoma who received at least one dose of an ICI. Pre-existing AID required a clinician-documented diagnosis before the first ICI dose. AID was considered clinically stable when there was no documented active exacerbation requiring initiation or escalation of systemic immunosuppressive therapy at ICI start. Patients were excluded for isolated autoimmune thyroid disease, concurrent participation in an interventional trial with protocol-mandated toxicity management, or insufficient follow-up or missing dates precluding ascertainment of PFS or OS. ICI regimens comprised anti-PD-1 monotherapy with nivolumab or pembrolizumab, and CTLA-4-containing treatment with nivolumab plus ipilimumab or ipilimumab alone. No anti-PD-L1 agent was administered.

### 4.3. Assessments

Best overall response was assessed according to RECIST 1.1 when available or by clinician-adjudicated radiological response. The retrospective dataset recorded treatment interruption attributed to worsening of the pre-existing AID and interruption for toxicity distinct from the baseline AID as patient-level binary variables. Complete event-level details, including organ, grade, onset date, corticosteroid dose and permanent-discontinuation status, were not uniformly available. The number of ICI cycles was not systematically recorded and was not analyzed.

### 4.4. Statistics

PFS was calculated from the first ICI dose to radiological progression or death from any cause, whichever occurred first; patients without an event were censored at the last disease assessment. OS was calculated from the first ICI dose to death from any cause, with living patients censored at the last follow-up. Kaplan–Meier methods were used to estimate PFS and OS, and unadjusted comparisons used the log-rank test.

Continuous and categorical baseline variables were compared using the Wilcoxon rank-sum test and Fisher exact test, respectively. ORR and DCR are reported with Wilson 95% CIs. Between-group effects are summarized as risk ratios with log-scale 95% CIs and two-sided Fisher exact *p* values.

The primary Cox proportional-hazards models evaluated the association between pre-existing AID and PFS or OS after adjustment for age per 10 years, sex, ICI regimen and M category. These covariates were prespecified on clinical grounds and were available in both cohorts. Stage IV was invariant. BRAF status was excluded because only two mutations were reported, both in the AID cohort, and metastatic-site burden was unavailable for controls. Proportional-hazards assumptions were assessed using scaled Schoenfeld residuals. Because dates of flare, de novo immune-related adverse events and initiation of additional immunosuppression were not consistently available, these variables were not modeled as time-dependent exposures. All tests were two-sided, with *p* < 0.05 considered statistically significant. Analyses were performed in R version 4.5.2 (R Foundation for Statistical Computing, Vienna, Austria).

## 5. Conclusions

ICI therapy may be considered for carefully selected patients with stage IV melanoma and clinically stable pre-existing AID. Treatment-related toxicity requires structured monitoring and multidisciplinary management. The adjusted PFS association should be interpreted cautiously, and the absence of an independent OS association argues against considering AID itself a favorable prognostic factor.

Prospective studies with standardized AID activity measures, complete toxicity ascertainment and longitudinal treatment data are needed to refine patient selection and management.

## Figures and Tables

**Table 1:** Baseline characteristics of the AID and control cohorts. *p* values were calculated using the Wilcoxon rank-sum test for age and Fisher exact tests for categorical variables. AID, autoimmune disease; AJCC, American Joint Committee on Cancer; ICI, immune checkpoint inhibitor; LDH, lactate dehydrogenase; n.a., not applicable. Shading identifies column headings and section labels only and does not encode statistical significance. The symbol *n* denotes the number of patients.

Characteristic	AID *n* = 41	Control *n* = 162	*p* Value
Age, median (range), years	54 (26–82)	66 (33–91)	<0.001
Female/male sex, *n* (%)	22 (53.7%)/19 (46.3%)	74 (45.7%)/88 (54.3%)	0.386
AJCC stage IV, *n* (%)	41 (100%)	162 (100%)	n.a.
M category, overall	3/14/22/2	36/33/74/19	0.037
M1a	3 (7.3%)	36 (22.2%)	
M1b	14 (34.1%)	33 (20.4%)	
M1c	22 (53.7%)	74 (45.7%)	
M1d	2 (4.9%)	19 (11.7%)	
M1c-M1d, *n* (%)	24 (58.5%)	93 (57.4%)	1.000
BRAF wild-type, *n* (%)	39 (95.1%)	162 (100%)	0.040
BRAF mutation, *n* (%)	2 (4.9%)	0	
LDH			
High	5 (12.2%)	22 (13.6%)	
Normal	31 (75.6%)	129 (79.6%)	
Missing	5 (12.2%)	11 (6.8%)	
ICI regimen			<0.001
Anti-PD-1 monotherapy	31 (75.6%)	162 (100%)	
CTLA-4-containing regimen	10 (24.4%)	0	

**Table 2:** Distribution of pre-existing autoimmune diseases in the AID cohort. Header shading identifies column headings only. The symbol *n* denotes the number of patients.

Autoimmune Disease	*n*
Psoriasis/psoriatic disease	6
Inflammatory arthritis	6
Systemic lupus erythematosus	2
Crohn disease	5
Ulcerative colitis/proctitis	10
Multiple sclerosis	6
Other immune-mediated conditions	6

Note: Two patients had more than one documented autoimmune diagnosis. For descriptive tabulation, each patient was assigned to one predominant category to avoid double counting.

## Data Availability

The raw data presented in this study are openly available in Zenodo: https://doi.org/10.5281/zenodo.21807242 (accessed on 12 August 2026).

## Supplementary Materials

The following supporting information can be downloaded at https://www.mdpi.com/article/10.3390/ijms27198832/s1.
