# Supplementary material for: Immune Checkpoint Inhibitor Data in Metastatic Melanoma with Pre-Existing Autoimmune Disease: A Real World Experience
Source: Int J Mol Sci. 2026 Oct 3;27(19):8832. doi: 10.3390/ijms27198832 (PMC13665204; doi:10.3390/ijms27198832)
Supplement: Supplementary file 1 [file ijms-27-08832-s001.zip › ijms-4529203-supplementary.pdf]

Supplementary Materials

Table S1. Selected published evidence on immune checkpoint inhibitors in patients with pre-existing autoimmune disease, including baseline treatment and efficacy or safety outcomes.

Study (Year)	N (AID patients)	Baseline immunosuppression or AID-directed treatment	Selected efficacy and safety outcomes
Menzies et al., Ann Oncol 2017 [6]	52	20/52 (38%) receiving systemic immunosuppression at ICI initiation	ORR 33%; median PFS 6.2 months (95% CI 4.2-8.2)
Xie et al., Autoimmun Rev 2020 (meta-analysis) [7]	619	Mixed (with and without IS)	Pooled ORR 30% (95% CI 22-39%)
Alexander et al., Ann Transl Med 2021 [8]	42	Not quantified	AID flare in 12/42 patients (29%); de novo irAEs in 9/42 (21%)
Fountzilas et al., HeCOG 2022 [9]	123	45/123 (36.6%) receiving AID-directed anti-inflammatory treatment; 26/123 (21.1%) receiving corticosteroids, alone or with other agents	In the NSCLC subgroup, baseline corticosteroids were associated with shorter PFS (unadjusted HR 2.78); irAE occurrence was associated with longer PFS (unadjusted HR 0.48)
Leonardi et al., JCO 2018 [11]	56	20% receiving immunomodulatory agents at ICI initiation	ORR 22%
Richter et al., Arthritis Rheumatol 2018 (Mayo) [12]	16	7/16 (43.8%) receiving immunosuppressive therapy or glucocorticoids at ICI initiation	irAEs in 6/16 patients; events managed with glucocorticoids and ICI discontinuation
Hoa et al., Cancer Immunol Immunother 2021 [13]	27	Yes (not quantified)	AID exacerbation rate 52%; flares occurred despite background immunosuppression
Plaçais et al., Ann Rheum Dis 2022 [14]	110	Not clearly reported	Better OS at 24 months vs controls
Sumimoto et al., PLoS One 2024 [15]	28	Ongoing AID-directed treatment reported; not equivalent to systemic immunosuppression	Pre-existing AID was associated with irAE occurrence (adjusted OR 2.52, 95% CI 1.08-5.86) and ICI discontinuation due to irAEs (adjusted OR 3.32, 95% CI 1.41-7.78)
Brown et al., JITC 2021 (combo anti-PD1 + IPI) [17]	55	Patients with and without baseline immunosuppression included	ORR 55%; median PFS 10 months; median OS 24 months. Baseline immunosuppression was associated with greater flare risk (OR 4.59; P = 0.03)
Tison et al., Arthritis Rheumatol 2019 [18]	112	24/112 (22%) receiving immunosuppressive therapy at ICI initiation	Median PFS 3.8 months with baseline immunosuppression versus 12 months without (P = 0.006)
Rakshit and Molina, J Thorac Dis 2020 (narrative review) [10]	Not applicable; no original cohort	Varies across the studies reviewed	Narrative synthesis of published cohorts; no independent pooled ORR, DCR or PFS estimate

Reference numbers correspond to the main manuscript. Cohorts differ in tumor type, autoimmune diagnosis and ICI regimen; estimates should not be compared directly. AID-directed treatment may include non-immunosuppressive agents. The narrative review is not an additional patient cohort. AID, autoimmune disease; CI, confidence interval; DCR, disease control rate; HR, hazard ratio; ICI, immune checkpoint inhibitor; irAE, immune-related adverse event; IS, immunosuppression;

NSCLC, non-small-cell lung cancer; OR, odds ratio; ORR, objective response rate; OS, overall survival; PFS, progression-free survival.

Table S2. Baseline characteristics and formal comparisons between the AID and control cohorts.

Characteristic	AID n = 41	Control n = 162	P value	Test
Age, median (range), years	54 (26-82)	66 (33-91)	<0.001	Wilcoxon rank-sum
Female/male sex	22 (53.7%)/19 (46.3%)	74 (45.7%)/88 (54.3%)	0.386	Fisher exact
AJCC stage IV	41 (100%)	162 (100%)	n.a.	Invariant
M1a/M1b/M1c/M1d	3/14/22/2	36/33/74/19	0.037	Fisher exact, 2 x 4
M1c-M1d	24 (58.5%)	93 (57.4%)	1.000	Fisher exact; OR 1.05 (0.52-2.10)
BRAF wild-type	39 (95.1%)	162 (100%)	0.040	Fisher exact
BRAF mutation	2 (4.9%)	0		
Anti-PD-1 monotherapy	31 (75.6%)	162 (100%)	<0.001	Fisher exact
CTLA-4-containing regimen	10 (24.4%)	0		

P values were calculated using the Wilcoxon rank-sum test for age and Fisher exact tests for categorical variables. AID, autoimmune disease; AJCC, American Joint Committee on Cancer; OR, odds ratio; n.a., not applicable.

Table S3. Exploratory Cox models for PFS and OS within the AID cohort.

Endpoint	Covariate	HR	95% CI	P value	N/events
PFS	>=3 metastatic sites	2.32	0.72-8.68	0.160	40/13
PFS	CTLA-4-containing vs anti-PD-1	1.36	0.38-4.22	0.609	40/13
OS	Age per 10 years	1.15	0.78-1.77	0.473	40/11
OS	>=3 metastatic sites	2.24	0.60-9.41	0.234	40/11
OS	CTLA-4-containing vs anti-PD-1	1.13	0.26-4.05	0.861	40/11

One patient with missing metastatic-site burden was excluded (n = 40). These analyses are exploratory. CI, confidence interval; HR, hazard ratio; OS, overall survival; PFS, progression-free survival.

Table S4. Cox proportional-hazards models for PFS and OS in the full cohort.

Endpoint	Model	Covariate	HR	95% CI	P value	N/events
PFS	Unadjusted	AID vs control	0.43	0.24-0.77	0.005	203/114
PFS	Adjusted	AID vs control	0.48	0.24-0.99	0.046	203/114
PFS	Adjusted	Age per 10 years	1.20	1.03-1.40	0.023	203/114
PFS	Adjusted	Male vs female	1.00	0.68-1.45	0.982	203/114
PFS	Adjusted	CTLA-4-containing vs anti-PD-1	1.51	0.46-4.94	0.492	203/114
PFS	Adjusted	M1b vs M1a	0.84	0.45-1.58	0.585	203/114
PFS	Adjusted	M1c vs M1a	1.57	0.95-2.59	0.081	203/114
PFS	Adjusted	M1d vs M1a	1.82	0.92-3.62	0.087	203/114
OS	Unadjusted	AID vs control	0.50	0.26-0.93	0.030	203/92
OS	Adjusted	AID vs control	0.63	0.29-1.34	0.232	203/92
OS	Adjusted	Age per 10 years	1.31	1.10-1.57	0.003	203/92
OS	Adjusted	Male vs female	0.80	0.53-1.21	0.290	203/92
OS	Adjusted	CTLA-4-containing vs anti-PD-1	1.21	0.32-4.61	0.775	203/92
OS	Adjusted	M1b vs M1a	0.73	0.34-1.58	0.430	203/92
OS	Adjusted	M1c vs M1a	2.01	1.12-3.60	0.019	203/92
OS	Adjusted	M1d vs M1a	2.82	1.32-6.02	0.007	203/92

Adjusted models included AID status, age per 10 years, sex, ICI regimen and M category. Reference categories were control cohort, female sex, anti-PD-1 monotherapy and M1a. CI, confidence interval; HR, hazard ratio; OS, overall survival; PFS, progression-free survival.

Table S5. Sensitivity analyses assessing the contribution of age to the association between AID and OS.

Model	Covariates	AID HR	95% CI	P value
Unadjusted	AID only	0.50	0.26-0.93	0.030
Age-adjusted	AID + age	0.63	0.33-1.21	0.169
Without age	AID + sex + regimen	0.46	0.22-0.96	0.038
Without age	AID + sex + regimen + M category	0.49	0.23-1.02	0.056
Fully adjusted	AID + age + sex + regimen + M category	0.63	0.29-1.34	0.232

Hazard ratios compare the AID cohort with controls. AID, autoimmune disease; CI, confidence interval; HR, hazard ratio; OS, overall survival.

Table S6. Comparative estimates of objective response rate and disease control rate.

Outcome	AID rate (95% CI)	Control rate (95% CI)	Risk difference (95% CI)	Risk ratio (95% CI)	Fisher P
ORR	46.3% (32.1-61.3)	32.7% (26.0-40.3)	13.6 pp (-2.5 to 30.0)	1.42 (0.95-2.11)	0.143
DCR	80.5% (66.0-89.8)	51.2% (43.6-58.8)	29.3 pp (12.9-41.3)	1.57 (1.27-1.94)	<0.001

Group-specific confidence intervals are Wilson intervals. Risk-difference confidence intervals use the Newcombe hybrid-score method; risk-ratio confidence intervals use the log scale. P values are from two-sided Fisher exact tests. AID, autoimmune disease; CI, confidence interval; DCR, disease control rate; ORR, objective response rate; pp, percentage points.

Table S7. Treatment interruptions related to autoimmune flare or other toxicity in the AID cohort.

Recorded variable	AID n = 41	Control cohort	Source-data interpretation
Treatment interruption attributed to flare of pre-existing AID	11 (26.8%)	Not applicable	Patient-level binary field
Treatment interruption for toxicity distinct from pre-existing AID	13 (31.7%)	Not systematically available	Patient-level binary field

These variables describe treatment interruption recorded at patient level and should not be interpreted as a complete event-level registry of all flares or de novo immune-related adverse events. AID, autoimmune disease.
